# Supplementary material for: A high-resolution mRNA expression time course of embryonic development in zebrafish
Source: eLife. 2017 Nov 16;6:e30860. doi: 10.7554/eLife.30860 (PMC5690287; doi:10.7554/eLife.30860)
Supplement: Supplementary file 6. [file elife-30860-supp6.zip › biolayout-clusters-files/Cluster233-genes.html]

Cluster233


# Cluster233: Genes

| Ensembl ID | Gene Name | Chr | Start | End | Biotype |
| --- | --- | --- | --- | --- | --- |
| ENSDARG00000100082 | AP3B1 | 21 | 6540249 | 6591403 | protein\_coding |
| ENSDARG00000077830 | borcs5 | 25 | 17824267 | 17828746 | protein\_coding |
| ENSDARG00000098853 | ehd1a | 14 | 46447388 | 46476820 | protein\_coding |
| ENSDARG00000018022 | msh2 | 12 | 24784887 | 24807278 | protein\_coding |
| ENSDARG00000038917 | rad50 | 9 | 54811561 | 54861231 | protein\_coding |
| ENSDARG00000035913 | yars | 19 | 31297393 | 31307959 | protein\_coding |
